# In Vitro Anti-Biofilm and Antibacterial Properties of *Streptococcus downii* sp. nov.

**DOI:** 10.3390/microorganisms9020450

**Published:** 2021-02-22

**Authors:** Maigualida Cuenca, María Carmen Sánchez, Pedro Diz, Lucía Martínez-Lamas, Maximiliano Álvarez, Jacobo Limeres, Mariano Sanz, David Herrera

**Affiliations:** 1 ETEP (Etiology and Therapy of Periodontal and Peri-Implant Diseases) Research Group, University Complutense of Madrid (UCM), 28040 Madrid, Spain; maiguacu@ucm.es (M.C.); marsan@ucm.es (M.S.); davidher@ucm.es (D.H.); 2 Medical-Surgical Dentistry Research Group (OMEQUI), Health Research Institute of Santiago de Compostela (IDIS), University of Santiago de Compostela (USC), 15705 Santiago de Compostela, Spain; pedro.diz@usc.es (P.D.); jacobo.limeres@usc.es (J.L.); 3 Clinical Microbiology, Microbiology and Infectology Group, Galicia Sur Health Research Institute, Hospital Álvaro Cunqueiro, Complejo Hospitalario Universitario de Vigo, Vigo, 36312 Galicia, Spain; lu_13128@hotmail.com (L.M.-L.); maximiliano.alvarez.fernandez@sergas.es (M.Á.)

**Keywords:** oral biofilm, *Streptococcus downii* sp. nov., caries, *Streptococcus mutans*, periodontal diseases, *Aggregatibacter actinomycetemcomitans*

## Abstract

The aim of this study was to evaluate the potential anti-biofilm and antibacterial activities of *Streptococcus downii* sp. nov. To test anti-biofilm properties, *Streptococcus mutans, Actinomyces naeslundii, Veillonella parvula, Fusobacterium nucleatum, Porphyromonas gingivalis,* and *Aggregatibacter actinomycetemcomitans* were grown in a biofilm model in the presence or not of *S. downii* sp. nov. for up to 120 h. For the potential antibacterial activity, 24 h-biofilms were exposed to *S. downii* sp. nov for 24 and 48 h. Biofilms structures and bacterial viability were studied by microscopy, and the effect in bacterial load by quantitative polymerase chain reaction. A generalized linear model was constructed, and results were considered as statistically significant at *p* < 0.05. The presence of *S. downii* sp. nov. during biofilm development did not affect the structure of the community, but an anti-biofilm effect against *S. mutans* was observed (*p* < 0.001, after 96 and 120 h). For antibacterial activity, after 24 h of exposure to *S. downii* sp. nov., counts of *S. mutans* (*p* = 0.019) and *A. actinomycetemcomitans* (*p* = 0.020) were significantly reduced in well-structured biofilms. Although moderate, anti-biofilm and antibacterial activities of *S. downii* sp. nov. against oral bacteria, including some periodontal pathogens, were demonstrated in an in vitro biofilm model.

## 1. Introduction

The oral cavity has one of the richest microbial communities within the human microbiome, with around 200 cultivable bacterial species and approximately 1000 phylotypes detected by 16S rRNA gene sequencing [1,2]. In healthy subjects, the majority of these species are commensal and maintain a symbiotic relation with the host, being the predominant phyla: Firmicutes, Proteobacteria, Actinobacteria, Bacteroidetes, and Fusobacteria [3]. Among Firmicutes the most abundant species belongs to *Streptococcus, Veillonella,* and *Lactobacillus* genera [4]. However, under specific environmental conditions, existing pathobionts may overgrow and become pathogenic, mainly by disturbing the homeostasis between the bacterial challenge and the host immune responses (dysbiosis) [5], which can also be associated with changes in microbial metabolism and/or shifts in bacterial diversity. Moreover, within the oral cavity, overgrow of microorganisms usually occurs forming highly structured poly-microbial communities, as biofilms, what may hinder the efficacy of host defenses and natural antimicrobial strategies.

Intervention studies have confirmed the etiological importance of biofilms by demonstrating that mechanical biofilm disruption is a key step in the effective management of biofilm-related oral conditions, caries, and periodontal diseases. In the treatment of the most aggressive/severe cases of periodontitis, more effective outcomes have been demonstrated when mechanical debridement has been supplemented with the adjunctive use of antibiotics [6] and antiseptics [7]. The use of antimicrobials, however, is controversial, due to the possible occurrence of secondary effects and the development of bacterial resistances [6,7,8,9], which calls for exploring alternative strategies.

One of these alternative strategies is to foster a health-associated microbiota that will transform the dysbiotic state into re-establishing homeostasis. One strategy in this direction has been the use of live microorganisms (probiotics), mainly bifidobacteria and lactobacilli, which have shown adjunctive benefits in the prevention and treatment of periodontal diseases [10,11,12,13,14,15,16,17,18,19,20]. This benefit has been attributed to the ability of these probiotics (i) to occupy a habitat, and thus reduce pathogen adhesion and colonization; (ii) to boost the host immune response; and (iii) since they have inherent antimicrobial activity [21]. Another strategy, seldom explored and investigated, has been the use of indigenous oral species, which will occupy and ecological niche, and thus will reduce the adhesion and growth of opportunistic pathogens and pathogenic species [22,23,24,25,26]. *Streptococcus* spp., such as *Streptococcus sanguinis*, *Streptococcus cristatus*, *Streptococcus salivarius,* or *Streptococcus mitis*, have shown their ability to inhibit the in vitro colonization of epithelial cells by *Aggregatibacter actinomycetemcomitans* [24,25], *S. mitis* showed antagonism in the adhesion of *Porphyromonas gingivalis* [23], and *Streptococcus dentisani*, isolated from caries-free individuals, showed growth inhibition of periodontal pathogens and against pathogens implicated in dental root infections, in pure culture [22,27,28]. Moreover, *S. dentisani*, attached to gingival cells in vitro, inhibits periodontal pathogens by competition, adherence, and displacement mechanisms [28]. Similarly, bifidobacteria may have the capacity of suppressing the growth of *P. gingivalis* by reducing key nutritional factor(s) in the environment [26].

Recently, derived from the analysis of the oral microbiota of patients with Down syndrome a novel species of bacteria of the *Streptococcus oralis* group, *Streptococcus downii* sp. nov., has been described [29]. By inhibition assays, *S. downii* sp. nov. has exhibited a potentially antimicrobial effect against the cariogenic bacteria *Streptococcus mutans* and against the periodontal pathogens *Veillonella parvula* and *A. actinomycetemcomitans* [29].

However, despite the fact that oral bacteria are organized in biofilms, most of these in vitro studies cited have investigated this antibacterial potential against bacteria in the planktonic state, rather than assessing this effect on biofilm models, thus mimicking a situation closer to what happens in vivo. Therefore, based on the results of individual species—species that determined the specific effect on *S. mutans*, *V. parvula* and *A. actinomycetemcomitans*, the purpose of this investigation was to assess (i) the potential anti-biofilm activity of *S. downii* sp. nov., either by inhibiting the growth of selected oral bacteria and/or by interfering with biofilm formation when growing on an in vitro biofilm model, and (ii) the potential antibacterial effects against oral bacteria in mature biofilms.

## 2. Materials and Methods

### 2.1. Isolates, Culturing, and Bacterial Growth Conditions

*S. downii* sp. nov. strain CECT 9732T, isolated from a supragingival dental biofilm sample of an individual with Down syndrome [29], and the reference bacterial strains *S. mutans* ATCC 25175, *V. parvula* NCTC 11810, *Actinomyces naeslundii* ATCC 19039, *F. nucleatum* DMSZ 20482, *A. actinomycetemcomitans* DSMZ 8324, and *P. gingivalis* ATCC 33277 were used to develop a multi-species biofilm model. Bacteria were cultured anaerobically (10% H_2_, 10% CO_2_, and balance N_2_) on blood agar plates (Blood Agar Oxoid No 2; Oxoid, Basingstoke, UK), supplemented with 5% (*v/v*) sterile horse blood (Oxoid, Basingstoke, UK), 5.0 mg mL^−1^ hemin (Sigma-Aldrich, St. Louis, MO, USA) and 1.0 mg mL^-1^ menadione (Merck, Darmstadt, Germany) for 72 h at 37 °C.

### 2.2. Anti-Biofilm Activity of S. downii sp. nov. in an in Vitro Biofilm Model

**Figure 1:**
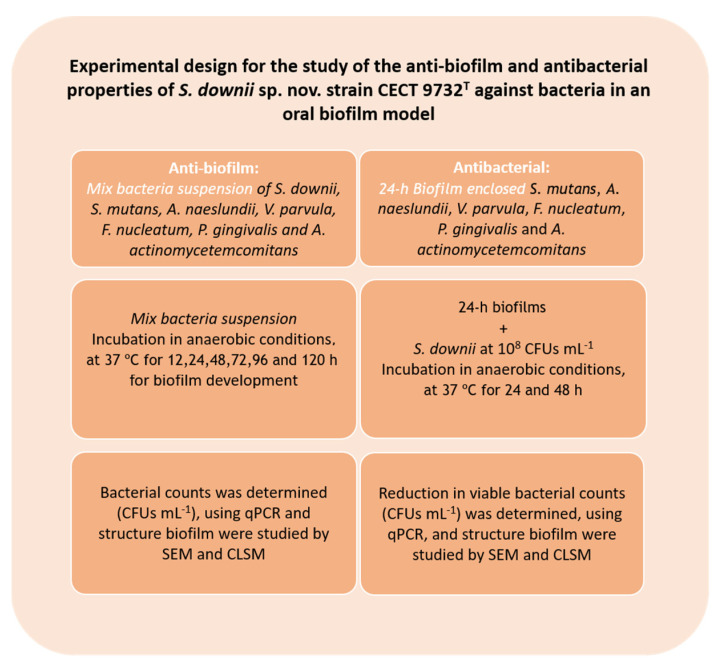
Scheme of the antibacterial assays carried out in the study. For abbreviations, see the text.

Figure 1 shows the experimental design followed for the study of the anti-biofilm properties of *S. downii* sp. nov. strain CECT 9732T against bacteria in an oral biofilm model.

#### 2.2.1. Biofilm Development

Biofilms were developed on hydroxyapatite (HA) discs in a static biofilm model, based on the model described by Sánchez et al. [30] with some modifications. In brief, representative colonies of all species were selected randomly and were grown on brain-heart infusion (BHI) medium (Becton, Dickinson and Company, Franklin Lakes, NJ, USA) supplemented with 2.5 g L^−1^ mucin (Oxoid, Basingstoke, UK), 1.0 g L^−1^ yeast extract (Oxoid, Basingstoke, UK), 0.1 g L^−1^ cysteine (Sigma-Aldrich, St. Louis, MO, USA), 2.0 g L^−1^ sodium bicarbonate (Merck, Darmstadt, Germany ), 5.0 mg L^−1^ hemin (Sigma-Aldrich, St. Louis, MO, USA), and 1.0 mg L^−1^ menadione (Merck, Darmstadt, Germany) and 0.25% (*v/v*) glutamic acid (Sigma-Aldrich, St. Louis, MO, USA), in anaerobic condition at 37 °C for 24 h. The bacterial growth was harvested at late exponential phase (measured by spectrophotometry), and a mixed bacterial suspensions in supplemented BHI medium were prepared in order to develop biofilms, using different starting concentrations, depending on their growth rate: 10^3^ colony forming units (CFU) mL^−1^ of *S. mutans*, 10^5^ CFU mL^−1^ of *V. parvula* and *A. naeslundii*, and 10^6^ CFU mL^−1^ of *F. nucleatum*, *A. actinomycetemcomitans,* and *P. gingivalis*.

Sterile HA discs [7-mm diameter and 1.8 (standard deviation—SD = 0.2) mm thickness (Clarkson Chromatography Products, Williamsport, PA, USA)] were placed in a multi-well tissue culture plate (Greiner Bio-one, Frickenhausen, Germany). Each well was inoculated with 1.5 mL of the mixed bacterial suspension, and to assess the anti-biofilm activity, treated wells were inoculated with 10^3^ CFU mL^−1^ of *S. downii* sp. nov. final concentration (recovered at late-exponential phase by centrifugation of an overnight culture and resuspended in fresh modified BHI medium). Control biofilms, not exposed to *S. downii* sp. nov., and treated ones were then incubated in anaerobiosis at 37 °C, for 12, 24, 48, 72, 96, and 120 h. Plates only containing culture media were also incubated to check for sterility. Three independent trials (on three different occasions) were carried out.

#### 2.2.2. DNA Isolation and Quantitative Polymerase Chain Reaction (qPCR)

Before the DNA isolation, discs were sequentially rinsed in 2 mL of sterile buffer saline (PBS) (immersion time per rinse, 10 s), three times, in order to remove non-adherent bacteria. Biofilms were then disrupted by vortex for 2 min in 1 mL of PBS. The DNA from the biofilm was extracted using an ATP Genomic DNA Mini Kit^®^ (ATP biotech. Taipei, Taiwan) according to the manufacturer’s recommendations. The bacterial 16S rRNA gene sequence was amplified by qPCR, according to the hydrolysis probes 5´nuclease method (Table 1). The qPCR amplification was performed in a total reaction mixture volume of 10 µL. The reaction mixtures contained 5 µL of 2× master mixture (LC 480 Probes Master; Roche, Mannheim, Germany), optimal concentrations of primers and probes (300, 300 and 300 nM, respectively, for *S. mutans*, *Streptococcus* spp., *A. naeslundii* and *P. gingivalis*; 750, 750, and 400 nM for *V. parvula*; 300, 300, and 200 nM for *A. actinomycetemcomitans*; and, finally, 600, 600, and 300 nM for *F. nucleatum*) and 2 µL of DNA from samples. The negative control was 2 µL of sterile water (Water PCR grade, Roche Mannheim, Germany). The samples were subjected to an initial amplification cycle of 95 °C for 10 min, followed by 40 cycles at 95 °C for 15 s and 60 °C for 1 min. Analyses was performed with a LightCycler^®^ 480 II thermocycler (Roche Mannheim, Germany). LightCycler^®^ 480 Multiwell Plates 384 and sealing foils were used (Roche Mannheim, Germany). Each DNA sample was analyzed in duplicate.

Quantification of bacteria by qPCR was based on standard curve. The correlation between Cq values and CFU mL^−1^ was automatically generated through the software (LC 480 Software 1.5; Roche Mannheim, Germany). Since the primers and probes targeting *Streptococcus* spp. detected all *Streptococcus* present in the sample, the number of *S. downii* sp. nov. was calculated by subtracting the number of *S. mutans*.

#### 2.2.3. Analysis of Biofilms by Confocal Laser Scanning Microscopy (CLSM) 

Biofilms grown on HA discs were stained with LIVE/DEAD^®^ BacLight^TM^ Bacterial Viability Kit solution (Molecular Probes B. V., Leiden, The Netherlands) at room temperature, with 1:1 fluorocromes ratio, in the dark for 10 min (SD = 1). The obtained fully hydrated biofilms were studied with a confocal laser scanning microscope, using a fixed-stage Ix83 Olympus inverted microscope, coupled to an Olympus FV1200 confocal system (Olympus; Shinjuku, Tokyo, Japan) with a ×63 water-immersion lenses (Olympus, Shinjuku, Tokyo, Japan). At least three separate and representative locations were selected from the HA discs covered with biofilm. With the use of a dedicated software (Olympus^®^ software (Olympus, Shinjuku, Tokyo, Japan) and Image analysis FIJI^®^ software (Image J v. 2.0.0-rc-65 /1.52b) a z-series of scans (xyz) of 0.5 µm thickness (8 bits, 1024 × 1024 pixels) were analyzed.

#### 2.2.4. Analysis of Biofilms by Scanning Electron Microscope (SEM)

Specimens were fixed in a solution at 4% paraformaldehyde and 2.5% glutaraldehyde for 4 h, at 4 °C. The discs were washed once in phosphate buffer saline (PBS) and another time in sterile water (immersion time per washed 10 min) and then dehydrated through a series of graded ethanol solutions (30, 50, 70, 80, 90, and 100%; immersion time per series 10 min). After that, specimens were critical point dried, sputter-coated with gold and analysed by electron microscopy JSM 6400 (JSM6400; JEOL, Tokyo, Japan) with a back-scattered electron detector and an image resolution of 25 kV.

### 2.3. Antibacterial Effect of S. downii sp. nov. Against Oral Species in an Already Established in Vitro Biofilm

Figure 1 presents the experimental design of the study on the antibacterial activity of *S. downii* sp. nov. strain CECT 9732T against bacteria, in an in vitro oral biofilm model.

#### 2.3.1. Biofilm Development

Biofilms were developed as described previously (Section 2.2.1), and a mixed bacterial suspensions in supplemented BHI medium were prepared in order to develop biofilms, containing 10^3^ CFU mL^−1^ of *S. mutans*, 10^5^ CFU mL^−1^ of *V. parvula* and *A. naeslundii*, and 10^6^ CFU mL^-1^ of *F. nucleatum*, *A. actinomycetemcomitans,* and *P. gingivalis*. HA discs (Clarkson Chromatography Products, Williamsport, PA, USA) and 1.5 mL of the bacterial suspension were placed in the multi-well tissue culture plate (Greiner Bio-one, Frickenhausen, Germany) and incubated at 37 °C in anaerobiosis for 24 h. After that, the 24 h developed biofilms were: (i) exposed to 1 mL of *S. downii* sp. nov. at 10^8^ CFU mL^−1^ in supplemented BHI medium or (ii) in the case of controls, exposed to 1 mL of fresh supplemented BHI medium. Biofilms were then incubated for additional 24 and 48 h at 37 °C in anaerobiosis. Three independent trials (on three different occasions) were carried out.

#### 2.3.2. Biofilm Analysis

The 24 h biofilms and the biofilms re-incubated another 24–48 h in the presence or not (controls) of *S. downii* sp. nov., were analysed. Confocal laser scanning microscopy and scanning electron microscopy (Section 2.2.3 and Section 2.2.4) displayed the biofilms structures and bacterial viability. Quantitative polymerase chain reaction was used to assess the effect of *S. downii* sp. nov. in bacterial load (amounts of each bacterium expressed as CFU mL^−1^; Section 2.2.2).

### 2.4. Statistical Analysis

The primary outcome variable was the count of viable bacteria present in the in vitro developed biofilms, expressed as viable CFU mL^−1^ of *S. downii* sp. nov., *S. mutans*, *V. parvula*, *A. naeslundii*, *A. actinomycetemcomitans*, *P. gingivalis,* and *F. nucleatum*. An experiment-level analysis was performed for each parameter of the study (*n* = 3). Shapiro–Wilk goodness-of-fit tests and distribution of data were used to assess normality. Data were expressed as means and standard deviations (SD).

In order to evaluate the impact of *S. downii* sp. nov. and the time of biofilm-development (up to 120 h) and their interaction with the primary outcome variable (counts expressed in CFU mL^−1^), a general linear model was constructed for each bacterial species, using the method of maximum likelihood and Bonferroni corrections for multiple comparisons. A *p*-value < 0.05 was considered statistically significant. A software package (IBM SPSS Statistics 21.0; IBM Corporation, Armonk, NY, USA) was used for all data analysis.

## 3. Results

### 3.1. Anti-Biofilm Activity of S. downii sp. nov. in an in Vitro Biofilm Model

**Figure 2:**
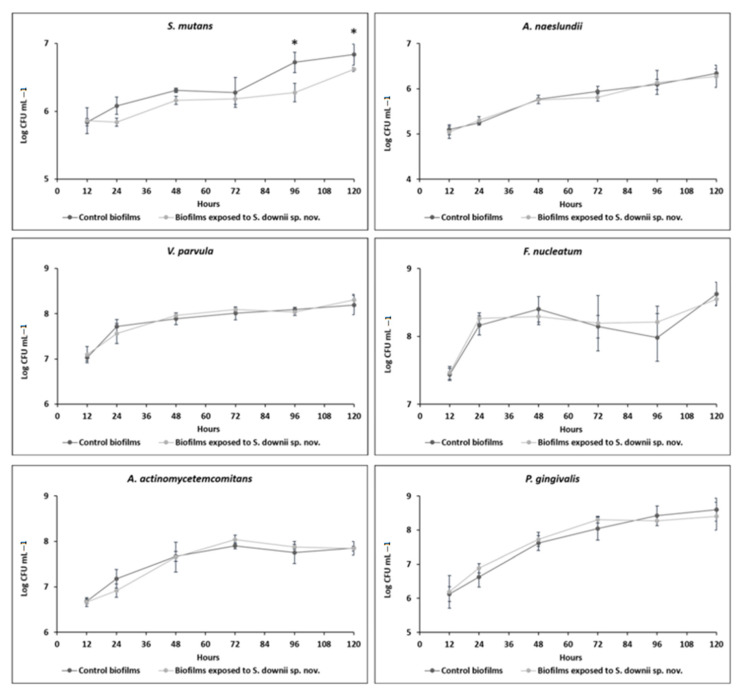
Kinetics of incorporation of the six selected bacterial strains in the biofilm [expressed as logarithm of Colony Forming Units per mL, (log CFU mL^−1^)] on the two biofilm modalities compared in the study: control biofilms (composed of *Streptococcus mutans, Veillonella parvula, Actinomyces naeslundii, Fusobacterium nucleatum, Aggregatibacter actinomycetemcomitans,* and *Porphyromonas gingivalis*) and experimental biofilms, incorporating also *Streptococcus downii* sp. nov. Analyses have been performed with quantitative polymerase chain reaction, in biofilms from 12 h to 120 h of incubation, using specific primers and probes directed to the 16S rRNA gene. *p < 0.005.

The impact of *S. downii* sp. nov. on the obtained multispecies biofilms was assessed from 12 to 120 h. Figure 2 depicts the kinetic profiles of the six bacterial species used in this in vitro biofilm model (strains *S. mutans*, *A. naeslundii*, *V. parvula*, *F. nucleatum*, *P. gingivalis,* and *A. actinomycetemcomitans*), quantified by qPCR, when forming biofilms with or without the presence of *S. downii* sp. nov. In biofilms exposed to *S. downii* sp. nov., it could be detected this bacterial species incorporated in the biofilm as early as 12 h of biofilm evolution and up to 120 h, with an average concentration of 2.7 × 10^7^ CFU mL^−1^ (SD = 1.5 × 10^7^).

In both obtained biofilms, with or without the presence of *S. downii* sp. nov, the six bacterial species were incorporated already after 12 h of incubation, and were present for up to 120 h. Only the growth of *S. mutans* was significantly different when *S. downii* sp. nov. was present in the biofilms (Figure 2). This impact was not observed in the early stages of biofilm formation, since at 12 h, both biofilms were similar (*p* > 0.05). From 24 to 72 h there was a clear trend, but without being statistically significant (*p* > 0.05 in all cases). However, differences were evident and statistically significant in the stationary phase (96 and 120 h) of the biofilm formation (*p* < 0.001, in both cases) (Figure 2). The kinetics of the rest of initial colonizers (*V. parvula* and *A. naeslundii*), and that of the periodontal pathogens (*F. nucleatum*, *A. actinomycetemcomitans,* and *P. gingivalis*) was not affected by the presence of *S. downii* sp. nov. during the 120 h of incubation (*p* > 0.05 in all cases).

**Figure 3:**
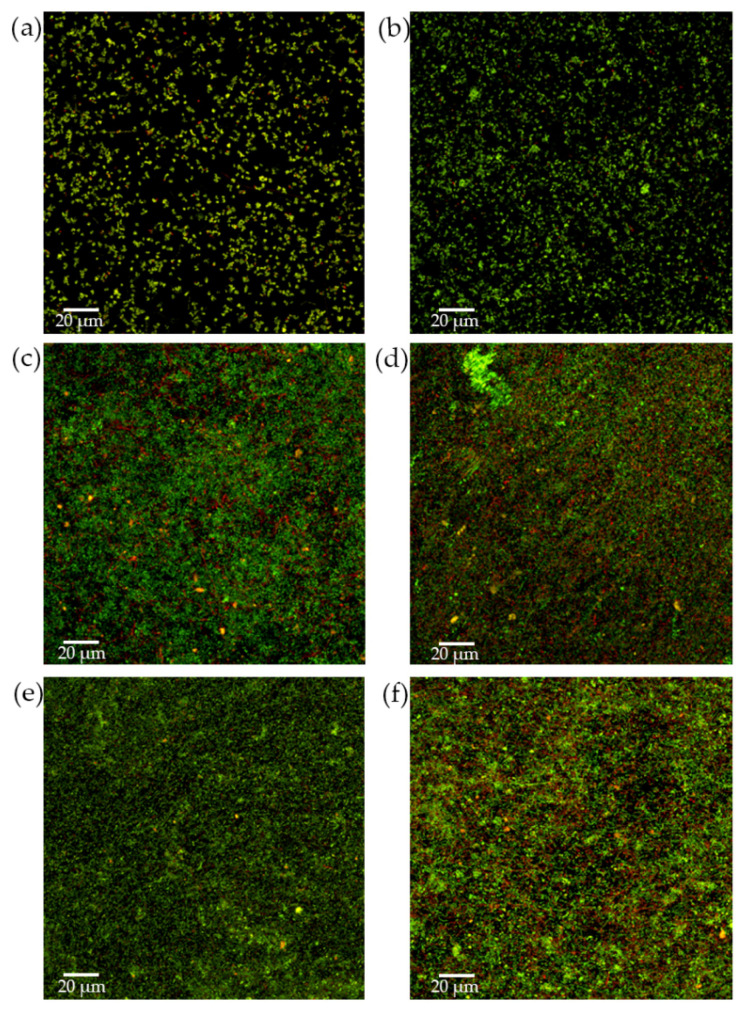
Confocal micrographs that represented a 2D maximum projection of the series along fixed axis of the control biofilms (**a**,**c**,**e**), composed by *Streptococcus mutans, Veillonella parvula, Actinomyces naeslundii, Fusobacterium nucleatum, Aggregatibacter actinomycetemcomitans,* and *Porphyromonas gingivalis*, and experimental biofilms, incorporating also *Streptococcus downii* sp. nov. (**b**,**d**,**f**), after 24 h (**a**,**b**), 72 h (**c**,**d**) and 120 h (**e**,**f**) of growth. LIVE/DEAD^®^ BacLight^TM^ Bacterial Viability Kit stain was used to assess the vitality of cells (live cells in green and dead cells in red color; yellowish corresponded to damage cells but still alive).

Biofilm structure and bacterial viability were studied by CLSM. During the initial growth phase, and after 24 h of incubation, biofilms containing *S. downii* sp. nov. depicted a well-structured bacterial community, similar to biofilms without *S. downii* sp. nov. (Figure 3a,b), with a live/dead ratio of 2.75 (SD = 0.2) for biofilms without *S. downii* sp. nov. and 2.4 (SD = 0.8) for biofilms containing *S. downii* sp. nov. This tendency was maintained during the exponential phase of biofilm development and, after 72 h, both modalities of biofilms showed a similar architecture, corresponding to a mature bacterial community (Figure 3c,d), with a live/dead ratio of 3.0 (SD = 0.1), for biofilms without *S. downii* sp. nov., and 2.7 (SD = 0.2), for biofilms containing *S. downii* sp. nov. However, and in agreement with the previously described qPCR data, in the stationary phase of biofilms (from 96 to 120 h, Figure 3e,f), it could be observed a higher percentage of dead cells in biofilms containing *S. downii* sp. nov., when compared with biofilms without *S. downii* sp. nov. The live/dead ratio was 2.0 (SD = 0.1) for biofilms without *S. downii* sp. nov. and 1.4 (SD = 0.4) for biofilms containing *S. downii* sp. nov.

**Figure 4:**
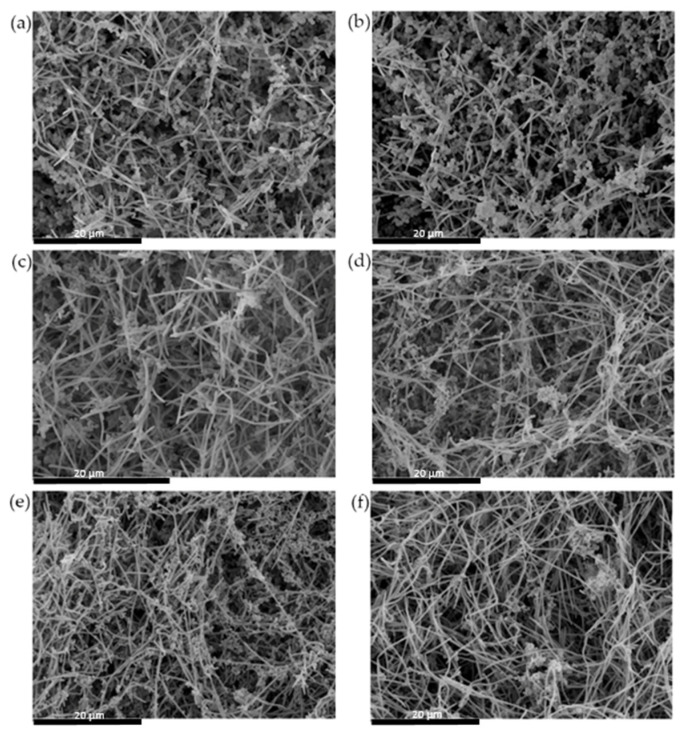
Scanning electron microscope images of the control biofilms (**a**,**c**,**e**), composed by *Streptococcus mutans, Veillonella parvula, Actinomyces naeslundii, Fusobacterium nucleatum, Aggregatibacter actinomycetemcomitans* and *Porphyromonas gingivalis*, and experimental biofilms, incorporating also *Streptococcus downii* sp. nov. (**b**,**d**,**f**), after 24 h (**a**,**b**), 72 h (**c**,**d**) and 120 h (**e**,**f**) of growth. A similar architecture of biofilms can be observed, in both presence and absence of *S. downii* sp. nov., with biofilms covering the disc surfaces with flat homogenous layers of cells, combined with bacterial clusters, showing channels inside the structure.

The analysis by SEM showed similar findings (Figure 4). After 120 h or growth, it would seem that the proportion of *S. mutans* forming chains of cocci was reduced in biofilms containing *S. downii* sp. nov., compared to grown biofilms without *S. downii* sp. nov. (Figure 4e,f).

### 3.2. Antibacterial Effect of S. downii sp. nov. Against Oral Species in an Already Established in Vitro Biofilm

**Figure 5:**
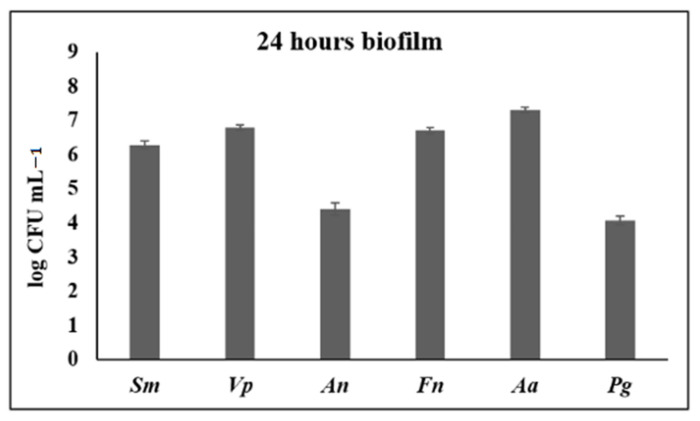
Analysis of 24-h biofilms by confocal laser scanning microscopy, showing a typical structure of biofilms, with bacteria in discontinuous microcolonies over hydroxyapatite surfaces, with a majority of live cells (live cells in green; dead cells in red; yellowish corresponded to damage cells but still alive); and counts (expressed as logarithm of colony forming units per mL, CFU mL^−1^) of *Streptococcus mutans* (*Sm*), *Veillonella parvula* (*Vp*), *Actinomyces naeslundii* (*An*), *Fusobacterium nucleatum* (*Fn*), *Aggregatibacter actinomycetemcomitans* (*Aa*), and *Porphyromonas gingivalis* (*Pg*).

After 24 h of incubation, well-structured biofilms were developed. The presence of the six inoculated bacterial species was confirmed by qPCR, and their live/dead ratio, measured by CLSM, was 2.0 (SD = 0.1) (Figure 5).

**Figure 6:**
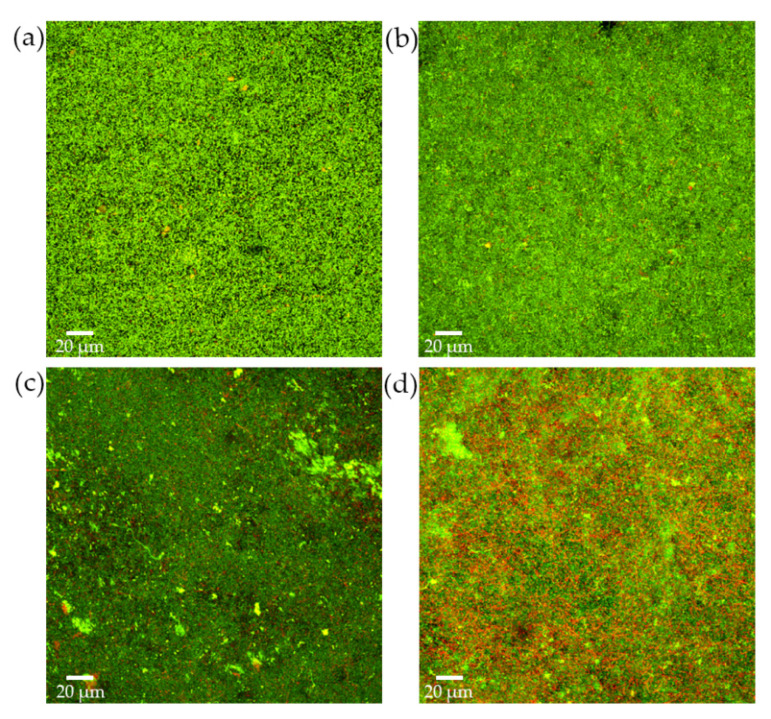
Analysis by confocal laser scanning microscopy of: (**a**,**b**) 24-h biofilms exposed for 24 h to 1 mL of brain heart infusion (BHI) medium (control biofilms) and to 1 mL of 10^8^ colony forming units (CFU) mL^−1^ of *S. downii* sp. nov. in fresh BHI medium (exposed biofilms), respectively; (**c**,**d**) 24-h biofilms exposed for 48 h to 1 mL of BHI medium (control biofilms) and to 1 mL of 10^8^ CFU mL^−1^ of *S. downii* sp. nov., in fresh BHI medium (exposed biofilms), respectively.

These biofilms were then exposed to 10^8^ CFU mL^−1^ of *S. downii* sp. nov. for 24 and 48 h and examined by CLSM (Figure 6). The obtained 24 h biofilms showed similar well-structured bacterial communities when compared those non-exposed versus exposed to *S. downii* sp. nov. (Figure 6a,b, respectively) and with live/dead ratio of 3.0 (SD = 0.3) and 4.2 (SD = 1.7), respectively. In those exposed to *S. downii* sp. nov., a thicker live biomass could be observed, possibly caused by the additional bacteria incorporated (Figure 6a,b). After 48 h of exposition, in spite of having a similar biofilm structure, the percentage of dead bacteria was significantly higher in the biofilms exposed to *S. downii* sp. nov. when compared with those without *S. downii* sp. nov. (Figure 6c,d; live/dead ratio of 1.1 (SD = 0.2) and 1.8 (SD = 0.2), respectively.)

**Figure 7:**
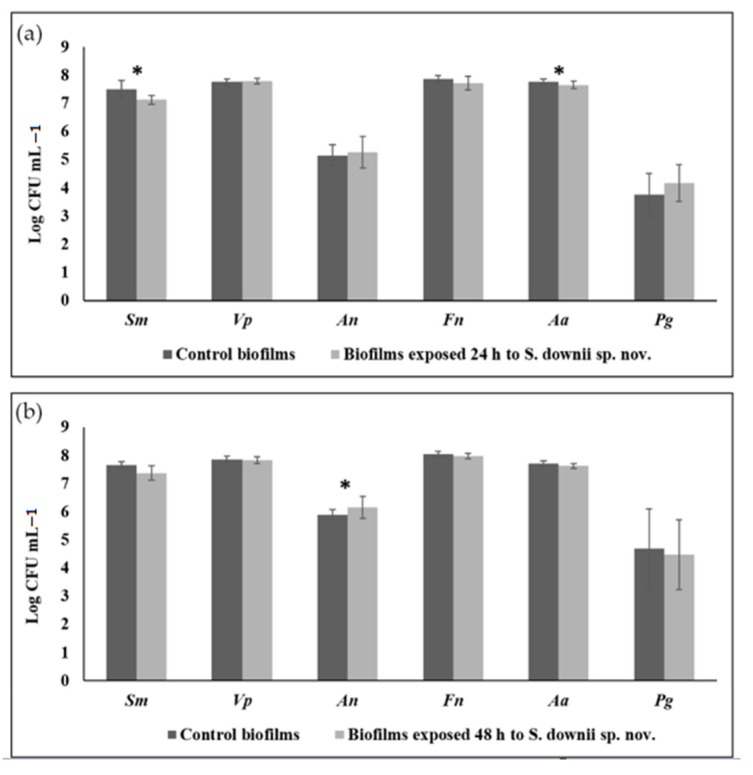
Counts (expressed as logarithm of colony forming units per mL, CFU/mL) of *Streptococcus mutans (Sm), Veillonella parvula (Vp), Actinomyces naeslundii (An), Fusobacterium nucleatum (Fn), Aggregatibacter actinomycetemcomitans (Aa)*, and *Porphyromonas gingivalis (Pg)* in biofilms using specific primers and probes directed to the 16S rRNA gene: (**a**) 24-h biofilms after contact with 10^8^ CFU/mL of *S. downii* sp. nov. for 24 h more; (**b**) 24-h biofilms after contact with 10^8^ CFU/mL of *S. downii* sp. nov. for 48 h more. * *p* < 0.005.

Figure 7 depicts the concentration (expressed in CFU mL^−1^) of the six inoculated bacteria when comparing those biofilms exposed and non-exposed to *S. downii* sp. nov. for 24 and 48 h. After 24 h of contact with *S. downii* sp. nov., the concentration of *S. mutans* (*p* = 0.019) and *A. actinomycetemcomitans* (*p* = 0.020) were significantly reduced, when compared to non-exposed biofilms (Figure 7a). After 48 h of contact, a similar tendency was observed, but with statistically significant higher concentrations for *A. naeslundii*, in exposed biofilms (*p* = 0.003) (Figure 7b). 

With longer time of incubation (from 24 to 48 h), the concentration of the selected bacteria increased in both types of biofilms, but for *P. gingivalis*, a significant increase (*p* = 0.018) was observed in non-exposed biofilms, while the increase in exposed biofilms was not statistically significant (*p* = 0.395). A similar trend was observed for *V. parvula* (*p* = 0.050 in non-exposed biofilms and *p* = 0.295 in exposed biofilms, respectively).

## 4. Discussion

The novel species *S. downii* sp. nov. has been recently isolated from a supragingival dental biofilm sample of an individual with Down syndrome [29]. The orofacial and skeletal developmental disturbances associated with Down syndrome contribute to frequent oral conditions and diseases in these patients, as periodontal diseases, malocclusion, mouth breathing, macroglossia, delayed teeth eruption, missing and malformed teeth, microdontia, diastema, and bruxism [31]. In spite of this, Down syndrome individuals have shown significantly lower prevalence of dental caries when compared with matched individuals without the syndrome [31]. In this regard, previous evidence has indicated antibacterial activity of *S*. *downii* sp. nov. against pathogens involved, not only in caries, but also in periodontal diseases, carried out by inhibition assays on solid culture medium [29].

Based on the previous findings, and in order to provide additional information, but with a model that better resembles what occurs in the oral cavity (bacteria organized in biofilms), the potential effect on oral health of *S. downii* sp. nov. was studied using a validated in vitro biofilm model. The present manuscript describes the in vitro antibacterial and anti-biofilm effects of *S. downii* sp. nov. when inoculated with oral bacteria included in a multispecies biofilm. Although there was no significant impact in the early stages of biofilm formation, during the stationary phase of biofilm growth, there was a significant reduction in the growth of *S. mutans* and *A. actinomycetemcomitans* that was evident with qPCR analysis.

This antibacterial activity on oral bacteria forming biofilms are in accordance with previous studies reported antibacterial activity of *S. mitis* group [23,24,25]. Similarly, other streptococci have demonstrated an impact on periodontal pathogens [22,28,32,33,34,35,36]. However, most of these in vitro studies were done using planktonic in vitro cultures, unlike the present study, carried out with an in vitro multispecies biofilm model. This difference may be relevant since not only translates closer the real bacterial growth in the oral cavity, but also demonstrates the antibacterial effect in an environment where these bacteria are more tolerant against antimicrobial agents. Another reason for choosing this in vitro biofilm model was to test the potential of the tested bacteria to adhere to oral surfaces, which can only be mimicked in such a model. Finally, the role of dental biofilms in the etiology of caries and periodontal diseases justifies the use of the present validated in vitro biofilm model [30], which was modified by changing the early colonizer *S. mutans* instead of *S. oralis*, since *S. mutans* has demonstrated a stronger association with both the onset and progression of dental caries [37,38,39].

When assessing the anti-biofilm capacity of *S. downii* sp. nov., an indigenous species of the oral cavity in subjects with Down syndrome, it was observed that it was able to effectively colonize the in vitro developed biofilms, without interfering with its structure and bacterial composition, since it did not affect the kinetics of colonization of secondary colonizers *(V. parvula* and *A. naeslundii*) or periodontal pathogens (*F. nucleatum, A. actinomycetemcomitans,* and *P. gingivalis*). However, its presence significantly interfered with the colonization kinetics of *S. mutans*, suggesting that the presence *S. downii* sp. nov. may control *S. mutans* levels, thus promoting health compatible biofilms or modifying their pathogenic potential [40].

Our findings agree with previous publications, which describe that some bacterial species, closely related to *S. downii* sp. nov., and isolated from caries-free subjects, exhibited antibacterial capacity against *S. mutans*. Bao et al. and Tong et al. [32,33] reported the inhibition of *S*. *mutans* by *Streptococcus oligofermentans,* due to the production of hydrogen peroxide, while Ogawa *et al*. [34], observed that *Streptococcus salivarius* inhibited the development of *S. mutans* biofilms, via bacteriocin production. More recently, the antibacterial capacity of *Streptococcus* A12 [35] and S. *dentisani* [22] against *S. mutans* was also described. Other studies reported that exogenous bacteria from the oral cavity could affect *S. mutans*, such as *Bifidobacterium* spp. isolated from human intestine [41], *Lactobacillus reuteri* strains, which were able to inhibit the growth of *S. mutans* in different degrees [42] or different *Lactobacillus* spp., which exhibits a notable degree of antagonism against *S. mutans* [41,43].

In regard to the ability to inhibit the formation and/or biofilm growth, as observed in the present research, this has been documented for other oral exogenous bacteria, including *Enterococcus faecium* WB2000, *Bifidobacterium adolescentis* SPM1005, or heat-inactivated *Bifidobacterium* BB12 [44,45,46]. Söderling et al. [47] observed that the probiotic strains *L. reuteri* SD2112 and *L. reuteri* PTA5289, *Lactobacillus rhamnosus* GG, and *Lactobacillus plantarum* 229v inhibited *S. mutans* biofilm formation on glass surfaces. Marttinem et al. [48] confirmed that *L. reuteri* ATCC PTA5289 could interfere with the adhesion of *S. mutans* to HA discs and inhibited biofilm formation. Lin et al. [49] refereed to the ability to inhibit *S. mutans* formation and biofilm growth on glass surfaces of other *Lactobacillus* strains, including *L. rhamnosus* HN001, *L. plantarum* ST-III, *Lactobacillus casei* strain Shirota, *L. casei* LC01, and *Lactobacillus paracasei* Lpc-37.

When assessing the antibacterial capacity of *S. downii* sp. nov. against a well-structured biofilm, this bacterial species was able to induced a significant lower concentrations of *S. mutans* (*p* = 0.019) and *A. actinomycetemcomitans* (*p* = 0.020), significant higher concentrations of *A. naeslundii* (*p* = 0.003) and prevented a statistically significant increment for *P. gingivalis* and *V. parvula* (*p* = 0.395 and p=0.295, respectively), compared to non-exposed biofilms. These results may suggest a potential probiotic effect of *S. downii* sp. nov. in the maintenance of oral homeostasis, decreasing the amounts of potential pathogenic species, and stimulating the growth of health-associated, potentially beneficial bacteria, such as *A. naeslundii*.

In periodontitis, both indigenous and exogenous bacterial strains have been in vivo and in vitro assessed, testing the hypothesis that they could help in the suppression of periodontal pathogens by competitive exclusion mechanisms and/or via the production of antimicrobial substances [28,36,50]. In vitro studies have shown positive results for different endogenous bacterial strains. *S. dentisani* showing inhibition of growth of periodontal pathogens and other pathogens implicated in root canals infections by the production of antimicrobial substances, although the study was conducted in planktonic in vitro cultures [22]. *S. mitis* demonstrated a successful antagonism in the adhesion of *P. gingivalis* [23], and also reduced the growth of *P. gingivalis* in presence of salivary bifidobacteria by reducing growth factor(s) from the environment [26]. Among exogenous strains, in vitro viability of *A. actinomycetemcomitans* was affected by human intestinal *Bifidobacterium* spp. [41]. *Bdellovibrio bacteriovorus* has been shown to significantly reduce the number of viable *A. actinomycetemcomitans*, both in planktonic and mono-species in vitro biofilm cultures [51]. However, it was observed that the efficiency decreased as the complexity of the model increased, i.e., with a model including six bacterial species (*P. intermedia, A. actinomycetemcomitans, P. gingivalis, F. nucleatum, S. mitis,* and *A. naeslundii*) [52].

This study presents clear limitations: an in vitro biofilm model consisting of six bacterial species has been used, and the effects of *S. downii* sp. nov. could be less evident in more diverse biofilms, closer to in vivo conditions; the application of the present findings could be limited due if there were relevant differences in the oral microbiota among Down syndrome subjects and other systemically healthy individuals and/or periodontitis patients [53,54], in whom *S. downii* sp. nov. may exhibit less capacity to colonize oral biofilms in vivo. The selected biofilm model consists in a common consortium of bacteria, including commensal bacteria and common periodontal pathogens strongly associated with periodontitis and caries, and the present study has shown, for the first time, antibacterial and antibiofilm activities of the tested novel species; thus, more studies are granted to better understand the mechanisms of action behind these findings.

## 5. Conclusions

In summary, the results of the present study have shown that an indigenous bacterial strain, *S. downii* sp. nov., had a moderate impact in oral biofilm formation and composition, as tested in a validated multi-species in vitro biofilm model. Firstly, it was shown that it was able to colonize the formed biofilms; secondly, and although the structural development was not affected by the presence of *S. downii* sp. nov., significantly lower amounts of *A. actinomycetemcomitans* (a periodontal pathogen, strongly associated with periodontitis) and of *S. mutans* (strongly associated with caries) were incorporated in the biofilms, in the presence of *S. downii* sp. nov. The present results, together with the previous findings showing α- and β-galactosidase activities of *S. downii* sp. nov. [29], which indicates that *S. downii* sp. nov. can use several saccharides as carbon source, including lactose, represents an advantage for its potential application as an oral probiotic [55].

## Figures and Tables

**Table 1:** Primers and probes used for quantification of genomic DNA from the target bacteria. Primers and probes were targeted against 16S rRNA gene (Obtained from Life Technologies Invitrogen (Carlsbad, CA, USA) and Roche (Roche Diagnostic GmbH; Mannheim, Germany)).

Bacteria	Sequence (5′–3′)	Length (bp)
*Streptococcus* spp.		
Forward	CAACGATACATAGCCGACCTGAG	97
Reverse	TCCATTGCCGAAGATTCC	
Probe	6FAM-CTCCTACGGGAGGCAGCAGTAGGGA-BBQ	
*S. mutans*		
Forward	GCCTACAGCTCAGAGATGCTATTCT	58
Reverse	GCCATACACCACTCATGAATTGA	
Probe	6FAM-TGGAAATGACGGTCGCCGTTATGAA-TMR	
*V. parvula*		
Forward	TGCTAATACCGCATACGATCTAACC	66
Reverse	GCTTATAAATAGAGGCCACCTTTCA	
Probe	6FAM-CTATCCTCGA+T+GCC+GA-BBQ	
*A. naeslundii*		
Forward	GGCTGCGATACCGTGAGG	103
Reverse	TCTGCGATTACTAGCGACTCC	
Probe	6FAM-CCCTAAAAGCCGGTCTCAGTTCGGAT-BBQ	
*P. gingivalis.*		
Forward	GCGCTCAACGTTCAGCC	67
Reverse	CACGAATTCCGCCTGC	
Probe	6FAM-CACTGAACTCAAGCCCGGCAGTTTCAA-TAMRA	
*A. actinomycetemcomitans*		
Forward	GAACCTTACCTACTCTTGACATCCGAA	80
Reverse	TGCAGCACCTGTCTCAAAGC	
Probe	6FAM-AGAACTCAGAGATGGGTTTGTGCCTTAGGG-TAMRA	
*F. nucleatum*		
Forward	GGATTTATTGGGCGTAAAGC	162
Reverse	GGCATTCCTACAAATATCTACGAA	
Probe	6FAM-CTCTACACTTGTAGTTCCG-TAMRA	

## Data Availability

Data available on request due to restrictions. The data presented in this study are available on request from the corresponding author. The data are not publicly available due to privacy issues.
